# Relationship between loneliness and blood glucose control in diabetes

**DOI:** 10.1186/s12889-020-09241-z

**Published:** 2020-07-20

**Authors:** Ewa Kobos, Alicja Szewczyk, Teresa Świątkowska, Tomasz Kryczka, Zofia Sienkiewicz

**Affiliations:** 1 Department of Development of Nursing, Social and Medical Sciences, Faculty of Health Sciences, Medical University of Warsaw, Żwirki i Wigury 61, 02-091 Warsaw, Poland; 2 Polish Federation for Education in Diabetology, Żegańska 21/23, 03-823 Warsaw, Poland; 3 The Children’s Memorial Health Institute, Clinic of Endocrinology and Diabetology, Al. Dzieci Polskich 20, 04-730 Warsaw, Poland; 4 Clinic of Endocrinology and Diabetology, Teaching Clinical Hospital, Marii Skłodowskiej Curie 9, 85-094 Bydgoszcz, Poland

**Keywords:** Loneliness, Diabetes mellitus, Metabolic control, Adult

## Abstract

**Background:**

The data of the International Diabetes Federation show that about 463 million people have diabetes. Better understanding of psychosocial aspects of life with this disease has become one of healthcare priorities in this group of patients. The aim of this study was to assess the relationships between loneliness and blood glucose control in diabetic patients.

**Methods:**

The study included 250 hospitalized patients with type 1 and 2 diabetes. The patients included in the study were those who had had diabetes for at least 1 year and received pharmacotherapy. Standardized Revised UCLA Loneliness Scale (R-UCLA) and an analysis of patient test results including 10 indicators of blood glucose control were used for data collection. Correlation analysis, i.e. Pearson’s linear correlation coefficient (r, parametric method), was used for hypothesis verification.

**Results:**

Less than one-fifth (16%) of the patients included in the study had higher loneliness index (based on the R-UCLA scale), and this loneliness index (total result) was significantly correlated with higher blood pressure. No significant correlations were demonstrated between loneliness and the other 9 indicators of blood glucose control.

**Conclusions:**

Systolic blood pressure was significantly correlated with loneliness in patients with diabetes. Further studies are needed to confirm these findings.

## Background

The data of the International Diabetes Federation show that about 463 million people have diabetes. It was estimated in 2019 that the number of diabetic patients aged between 65 and 99 years was 135.6 million (19.3%) [1]. Data from the National Health Fund estimate that there were 2.55 million of adult patients with diabetes in 2014 in Poland (4-year prevalence), and this number increased to 2.86 million in 2018. Diabetic individuals accounted for 9.1% of the adult Polish population in 2018 [2]. In the light of the growing global population of diabetic patients, better understanding of psychosocial aspects of life with this disease is becoming one of healthcare priorities in this group of patients [3].

The issue of loneliness in diabetic patients has been studied in various aspects. Determination of relationships between loneliness at an older age and metabolic biomarkers and vascular diseases was one of the aims of the research. It was found that the risk of increased levels of three out of four assessed metabolic biomarkers: glycated hemoglobin (HbA1c), body mass index (BMI), and metabolic burden [4], was increased by 39–71% in lonely elderly individuals. The mean incidence of type 2 diabetes mellitus was significantly higher in the group of lonely individuals [5].

Another aim of the study was to assess the relationship between loneliness and health problems. It was shown that individuals experiencing high levels of loneliness are more likely to develop chronic diseases, including diabetes mellitus and high cholesterol levels [6], and that these individuals are at particularly increased risk of diabetes [7–9]. It was found that treatment due to diabetes or hypertension was an important predictive factor for loneliness [10].

An analysis of loneliness as a factor affecting treatment in pre-diabetic and T2DM patients showed that these patients report the sense of isolation and loneliness even in the presence of social support [11]. An assessment of long-term blood glucose control showed that loneliness was associated with HbA1c levels due to the interaction between diet and social relationships [12], while no relationship was found between loneliness and fear of hypoglycemia in patients with type 1 diabetes mellitus [13]. We also assessed the relationship between loneliness and inflammatory/neuroendocrine responses to acute stress in elderly patients with T2DM. The authors of the study showed that loneliness was associated with disturbed responses to stress in diabetic patients, which may partly result from disregulation of the inflammatory and neuroendocrine systems [14]. An assessment of peer support from the perspective of adults with type 1 diabetes revealed a conviction that diabetes is present in all aspects of patients’ lives. The patients were unsatisfied with social relations in terms of being on one’s own, lack of connectedness and communality and lack of feeling understood [15].

Despite limited literature data on the role of loneliness in the treatment of diabetes, it was found that both these factors are interrelated. Diabetic complications lead to reduced physical activity and, consequently, limited social interactions. The disease also affects marriage, family and friendly relationships, which may contribute to loneliness. Conversely, systemic inflammation induced by stress in individuals experiencing loneliness is a risk factor for poor diabetes-related health outcomes [16].

To our knowledge, no study has yet assessed whether there is a relationship between loneliness and indicators of metabolic compensation achieved by adult patients with diabetes. We proposed a hypothesis that loneliness is a negative psychosocial factor, which makes it difficult for diabetic patients to achieve adequate blood glucose control. We investigated the relationship between loneliness and 10 indicators of metabolic compensation to verify this hypothesis. The aim of this study was to assess the relationships between loneliness and metabolic control in patients with diabetes mellitus.

## Methods

### Participants

This cross-sectional study was conducted in a group of 250 adult patients admitted to departments specialized in the treatment of diabetes mellitus in 6 hospitals between February and June 2019. Patients with at least 1-year history of diabetes, who received pharmacotherapy, were able to complete the questionnaire, and gave their oral consent to participate in the study were included in the research. The study was conducted on the first or second day of the patient’s stay in the ward. After receiving information on the purpose of the study and consenting to participate in the project, the patients completed the questionnaire on sociodemographic and clinical data, as well as the R-UCLA scale. Then, a person responsible for data collection included indicators of metabolic control in a table designed for this purpose.

A sociodemographic questionnaire was designed for the purposes of our study to collect sociodemographic and clinical data (Additional file 1). The questionnaire included sociodemographic data such as: age, sex, marital status, education, place of residence, professional status; and clinical data such as: disease duration, type of diabetes, type of treatment used, chronic diabetic complications, and the reason for hospital admission.

### Loneliness scale (R-UCLA)

Data on loneliness were assessed using a Polish version of standardized R-UCLA [17]. The original version of the scale was developed by Russell et al. [18]. The R-UCLA scale contains 20 items. Respondents were asked to indicate how often they experienced certain situations by rating each item on a 4-point Likert scale. The maximum score was 80, and the minimum score was 20. The higher the score, the higher the level of loneliness. According to Perry’s loneliness classification, a score between 65 and 80 indicates a high degree of loneliness, 50–64 – a moderately high degree of loneliness, 35–49 – a moderate degree of loneliness, and 20–34 – a low degree of loneliness [19].

### Indicators of metabolic control in diabetes

Criteria recommended by the Polish Diabetes Association were used for the assessment of metabolic compensation [20]. The values of clinical indicators were obtained from patient medical records. The following laboratory findings were evaluated: HbA1c (%), total cholesterol (mg/dL), HDL (mg/dL), LDL (mg/dL), triglicerides (mg/dL), non-ADL (mg/dL), LDL-C (mg/dL). Blood pressure, body weight and height were measured; BMI was calculated.

### Statistical analyses

The normality of distribution was assessed using the Shapiro-Wilk test. Homogeneity of variance was assessed using the Levene’s test. Hypotheses were verified using correlation analysis: Pearson’s linear correlation coefficient (parametric method). A *p*-value < 0.05 was considered statistically significant. Elements of descriptive statistics, such as mean, standard deviation, median, minimum and maximum values, were used for the assessment of metabolic control indicators.

## Results

The study included 250 patients aged between 18 and 94 years with type 1 and 2 diabetes. Mean age was 57.9 years (SD = 17.4). There were 54% of men, 29% of single, and 71% of non-single patients in the study group. Higher education was reported by 15% of respondents; 35% of respondents were professionally active. Type 2 diabetes was reported for 70% of patients; mean disease duration in the study group was 12.14 years (SD = 9.54); 26% of patients received antidiabetic medications; 24% of patients received both medications and insulin. Chronic complications occurred in 54% of patients. A total of 54% of patients were admitted to the department due to high glucose levels.

The mean score obtained by diabetic patients in the R-UCLA scale (the highest possible score was 80) was 38.22 (SD = 11.55). Low levels of loneliness were shown for 47% of patients, whereas very high levels were detected in 3% of respondents (Table 1).

**Table 1:** The sense of loneliness in the study group

Degrees of loneliness	%	M	SD	Med.	Min.	Max.
low degree (20–34)	47	38.22	11.55	35	23	76
moderate degree (35–49)	37					
moderately high degree (50–64)	13					
severe high degree (65–80)	3					

*M* mean, *SD* standard deviation, *Med*. median, *Min*. minimum, *Max*. maximum

A total of 80.8% of patients in the study group failed to meet the criteria of adequate blood glucose control for HbA1c < 7%, while 71.2% of patients failed to meet the BMI < 25 kg/m^2^ criterion (Table 2).

**Table 2:** Study participant characteristics - criteria of adequate blood glucose control

Criteria of adequate blood glucose control	Met criterion		
	n	Yes	No
BMI < 25 kg/m^2^	241	25.2%	71.2%
Systolic blood pressure < 130 mm/Hg	242	38.4%	58.4%
Diastolic blood pressure < 80 mm/Hg	242	32.4%	64.4%
HbA1c < 7%	236	13.6%	80.8%
Total cholesterol < 175 mg/dL	233	52.0%	41.2%
HDL cholesterol > 40 mg/dL [in men] and > 50 mg/dL [in women]	229	44.8%	46.8%
LDL cholesterol < 70 mg/dL	220	20.8%	67.2%
Triglyceride < 150 mg/dL	228	56.8%	34.4%

A positive correlation was shown between the sense of loneliness and systolic blood pressure (*r* = 0.165; *p* = 0.010). Patients with a higher degree of loneliness had increased systolic blood pressure (Table 3). No significant correlation was found between loneliness and the number of met criteria for adequate blood glucose control (*r* = − 0.052; *p* = 0.425).

**Table 3:** Correlations between loneliness and indicators of adequate blood glucose control

Criteria of adequate blood glucose control	Loneliness	
BMI	*r* = − 0.015	*p* = 0.815
Systolic blood pressure	*r* =v 0.165	***p*** **= 0.010**
Diastolic blood pressure	*r* = 0.083	*p* = 0.200
HbA1c	*r* = −0.090	*p* = 0.168
Total cholesterol	*r* = − 0.007	*p* = 0.907
HDL cholesterol	*r* = 0.041	*p* = 0.535
LDL cholesterol	*r* = − 0.012	*p* = 0.853
Triglyceride	*r* = 0.008	*p* = 0.898
non-HDL cholesterol *(n = 86)*	*r* = − 0.169	*p* = 0.119
LDL-C (*n = 52)*	*r* = − 0.042	*p* = 0.763

*r* Pearson’s correlation coefficient, *p* statistical significance, *n* number of participants

In the study group, diastolic blood pressure was significantly higher in men (*p* = 0.049), while HDL cholesterol was significantly higher among women (*p* = 0.013) (Table 4).

**Table 4:** Indicators of adequate blood glucose control by gender of study participants’

Variables	Man		Woman		t	df	p
	M	SD	M	SD			
BMI (kg/m^2^)	28.72	5.82	28.76	6.55	−0.049	239	0.960
Systolic blood pressure (mm/Hg)	134.83	19.59	134.06	19.87	0.305	240	0.760
Diastolic blood pressure (mm/Hg)	82.73	11.89	79.69	12,02	1.970	240	**0.049**
HbA1c (%)	9.15	2.40	9.25	2.58	−0.317	234	0.751
Total cholesterol (mg/dl)	173.93	61.68	175.94	59.66	−0.251	231	0.801
HDL cholesterol (mg/dl)	44.34	16.07	50.07	18.84	−2.480	227	**0.013**
LDL cholesterol (mg/dl)	93.02	39.57	96.08	44.19	−0.541	218	0.588
Triglyceride (mg/dl)	165.77	130.24	159.40	134.41	0.362	226	0.717
non-HDL cholesterol (mg/dl)	135.14	62.29	123.71	58.03	0.877	84	0.382
LDL-C (mg/dl)	117.25	94.39	101.73	61.27	0.703	50	0.485

*M* mean, *SD* standard deviation, *t* Pearson’s correlation coefficient, *df* degrees of freedom, *p* statistical significance

A significant, negative correlation between age and systolic/diastolic blood pressure was found in the study group. The younger the patients, the higher the systolic and diastolic blood pressure (Table 5).

**Table 5:** Systolic and diastolic blood pressure values by age of study participants’

Age	Systolic blood pressure (mm/Hg)		Diastolic blood p(mm/Hg)	
	*r* = − 0.128	*p* = **0.045**	*r* = − 0.1224	*p* = **0.057**

*r* Pearson’s correlation coefficient, *p* statistical significance

## Discussion

Diabetes mellitus is a common chronic disease in adults, and its incidence is likely to increase in the future [1]. Therefore, it is important to better understand everyday experiences, well-being and psychosocial functioning of individuals with this disease [3].

As mentioned in the introduction, there seems to be a relationship between loneliness and achieved therapeutic objectives in diabetes. Diabetes may lead to loneliness and, vice versa, loneliness may be a risk factor of poor treatment outcomes in diabetes [16]. We assessed the relationships between loneliness and metabolic control indicators. Available studies indicate that HbA1c and BMI were the most common metabolic control indicators assessed in relation to loneliness [12, 14, 21, 22]. An assessment of the relationship between loneliness and treatment adherence in diabetic patients showed a correlation between loneliness and postprandial blood glucose levels in these patients. Similarly to our study, no correlation was shown between HbA1c and BMI [21]. Also, no relationship was demonstrated between loneliness and these indicators in a study assessing the severity of already impaired response to stress in T2DM patients [14]. Although the relationship between loneliness and long-term control of glucose levels assessed based on HbA1c was not confirmed by Niemcryk [22], the importance of loneliness in T2DM patients in terms of following a healthy diet was confirmed during an attempt to identify predictors of long-term blood glucose control [12]. Although loneliness was positively correlated with increased HbA1c and BMI in the group of elderly patients, including those with diabetes, no such a correlation was found in our study. This study showed systolic hypertension in the total cohort (*n* = 466, 43 patients with T2DM) and both the lonely and non-lonely groups [5]. In our study, 58.4% of patients failed to meet the criterion of systolic blood pressure < 130 mmHg; individuals with higher degree of loneliness had higher systolic blood pressure. O’Luanaigh et al. and our study also failed to confirm the correlation between loneliness and lipid profile [5].

Clinical practice guidelines recommend a management strategy that targets multiple comorbid conditions, including hyperglycemia, hypertension, and dyslipidemia, to prevent diabetes-related complications [20]. The compliance observed in the population of patients with diabetes who received oral hypoglycemic agents, antihypertensives or statins at least once a day was not optimal [23, 24]. A review of studies found that among patients with diabetes, hypertension, and dyslipidemia, only 59% had medication possession ratio ≥ 80% [25]. Kusaslan Avci [21] and Kretchy [26] showed that loneliness associated with T2DM was significantly correlated with poor medication adherence. Ho and colleagues found that nonadherence to oral antidiabetic drugs, antihypertensives, or statins was related to increased A1C levels, higher systolic and diastolic blood pressure, and higher low-density lipoprotein cholesterol levels [27]. As this study showed significant association between loneliness and blood pressure, it is important to install positive attitude and lifestyle measures to tackle this lethal combination to reduce the risk of cardiovascular events among those exhibiting loneliness [28].

Our data indicate that there are no rationale for the hypothesis on a relationship between loneliness and indicators of blood glucose control in diabetes. Only higher systolic blood pressure was correlated with increased loneliness in the study population. Literature review indicates that data allowing for comparison of these findings are missing. Therefore, our results should be considered preliminary and further studies are needed to confirm these findings. These studies are important to enable a more comprehensive review of the psychosocial implications of living with diabetes.

Our study has some limitations. It was conducted only in a group of patients admitted to hospital, and thus with indications for hospitalization. Since increased glucose levels were the reason for admission in half of participants, it may be assumed that blood glucose control was unsatisfactory already at admission in this group of patients. In order to determine the scale of the problem, it is worth extending further research to include a group of patients reporting for visits to primary care outpatient clinics and diabetes outpatient clinics. No cognitive assessment of patients was performed. The study included patients who understood and could answer the questions in the questionnaire. Another limitation of this study was the fact that it was not verified whether the patients who were not in a relationship lived alone. We used a small sample and convenient selection, which means that only available patients were included in the study.

## Conclusions

This study contributes to determining loneliness in patients with diabetes in literature and shows the relationships between loneliness and blood glucose control. Less than one-fifth of the patients included in the study had high loneliness index. Systolic blood pressure was significantly correlated with loneliness in patients with diabetes. Our findings are an introduction to further studies in this area.

## Supplementary information

**Additional file 1.** Sociodemographic and clinical data questionnaire.

## Data Availability

The datasets generated and/or analyzed during the current study are not publicly available due to confidentiality, but data is accessible from the corresponding author on reasonable request.

## Abbreviations

DM: Diabetes mellitus
T2DM: Type 2 diabetes mellitus
HbA1c: Glycated hemoglobin
BMI: Body mass index

## References

[1] IDF DIABETES ATLAS Ninth edition 2019. International Diabetes Federation. https://diabetesatlas.org/upload/resources/material/20200106_152211_IDFATLAS9e-final-web.pdf (2019). Accessed 10 Mar 2020.

[2] Raport. NFZ o zdrowiu. Cukrzyca. Centrala Narodowego Funduszu Zdrowia, Departament Analiz i Strategii. Warszawa 2019. https://zdrowedane.nfz.gov.pl/pluginfile.php/205/mod_resource/content/4/nfz_o_zdrowiu_cukrzyca.pdf (2019). Accessed 10 Mar 2020.

[3] Nefs G, Bot M, Browne JL (2012). Diabetes MILES - the Netherlands: rationale, design and sample characteristics of a national survey examining the psychosocial aspects of living with diabetes in dutch adults. BMC Public Health.

[4] Shiovitz-Ezra S, Parag O (2019). Does loneliness ‘get under the skin’? Associations of loneliness with subsequent change in inflammatory and metabolic markers. Aging Ment Health.

[5] O'Luanaigh C, O'Connell H, Chin AV (2012). Loneliness and vascular biomarkers: the Dublin healthy ageing study. Int J Geriatr Psychiatry.

[6] Richard A, Rohrmann S, Vandeleur CL (2017). Loneliness is adversely associated with physical and mental health and lifestyle factors: results from a Swiss national survey. PLoS One.

[7] Foti SA, Khambaty T, Birnbaum-Weitzman O, et al. Loneliness, cardiovascular disease, and diabetes prevalence in the hispanic community health study/study of latinos sociocultural ancillary study. J Immigr Minor Health. 2020;22(2):345–52. 10.1007/s10903-019-00885-7 PMC6783350 30963348

[8] Christiansen J, Larsen FB, Lasgaard M (2016). Do stress, health behavior, and sleep mediate the association between loneliness and adverse health conditions among older people?. Soc Sci Med.

[9] Brinkhues S, Dukers-Muijrers NHTM, Hoebe CJPA (2017). Socially isolated individuals are more prone to have newly diagnosed and prevalent type 2 diabetes mellitus—the Maastricht study. BMC Public Health.

[10] Grover S, Verma M, Singh T (2019). Loneliness and its correlates amongst elderly attending non-communicable disease rural clinic attached to a tertiary care Centre of North India. Asian J Psychiatr.

[11] McConatha JT, Kumar VK, Raymond E, Akwarandu A (2020). Cultural dimensions of diabetes management: a qualitative study of middle eastern immigrants in the U.S. J Cross Cult Gerontol.

[12] Sarkadi A, Rosenqvist U (2001). Field test of a group education program for type 2 diabetes: measures and predictors of success on individual and group levels. Patient Educ Couns.

[13] Nefs G, Bevelander S, Hendrieckx C (2015). Fear of hypoglycaemia in adults with type 1 diabetes: results from diabetes MILES - the Netherlands. Diabet Med.

[14] Hackett RA, Poole L, Hunt E (2019). Loneliness and biological responses to acute stress in people with type 2 diabetes. Psychophysiology.

[15] Joensen LE, Filges T, Willaing I (2016). Patient perspectives on peer support for adults with type 1 diabetes: a need for diabetes-specific social capital. Patient Prefer Adherence.

[16] Petitte T, Mallow J, Barnes E (2015). A systematic review of loneliness and common chronic physical conditions in adults. Open Psychol J.

[17] Kwiatkowska MM, Rogoza R, Kwiatkowska K (2017). Analysis of the psychometric properties of the revised UCLA loneliness scale in a polish adolescent sample. Curr Issues Pers Psychol.

[18] Russell D, Peplau LA, Cutrona CE (1980). The revised UCLA loneliness scale: concurrent and discriminant validity evidence. J Pers Soc Psychol.

[19] Perry G (1990). Loneliness and coping among tertiary level adult cancer patients in the home. Cancer Nurs.

[20] Araszkiewicz A, Bandurska-Stankiewicz E, Budzyński A, et al. 2019 Guidelines on the management of diabetic patients. A position of Diabetes Poland. Clin Diabetol. 2019;8:1–95.

[21] Kusaslan AD (2018). Evaluation of the relationship between loneliness and medication adherence in patients with diabetes mellitus: a cross-sectional study. J Int Med Res.

[22] Niemcryk SJ, Speers MA, Travis LB (1990). Psychosocial correlates of hemoglobin Alc in young adults with type I diabetes. J Psychosom Res.

[23] Yang Y, Thumula V, Pace PF (2009). Predictors of medication nonadherence among patients with diabetes in Medicare part D programs: a retrospective cohort study. Clin Ther.

[24] Devaraj NK, Mohamed M, Hussein N (2017). Prevalence, factors influencing and knowledge about adherence to lipid-lowering therapy among hyperlipidemia patients. Med J Malaysia.

[25] Cramer JA, Benedict A, Muszbek N (2008). The significance of compliance and persistence in the treatment of diabetes, hypertension and dyslipidaemia: a review. Int J Clin Pract.

[26] Kretchy IA, Koduah A, Ohene-Agyei T, Boima V, Appiah B (2020). The association between diabetes-related distress and medication adherence in adult patients with type 2 diabetes mellitus: a cross-sectional study. J Diabetes Res.

[27] Ho PM, Rumsfeld JS, Masoudi FA (2006). Effect of medication nonadherence on hospitalization and mortality among patients with diabetes mellitus. Arch Intern Med.

[28] Sook LW, Sablihan NI, Ismail S (2019). Factors associated with the level of physical activities among non-academic staffs in the Faculty of Medicine and Health Sciences of a public university in Selangor, Malaysia. Mal J Med Health Sci.

