# Supplementary material for: Relationship between loneliness and blood glucose control in diabetes
Source: BMC Public Health. 2020 Jul 20;20:1140. doi: 10.1186/s12889-020-09241-z (PMC7372778; doi:10.1186/s12889-020-09241-z)
Supplement: Supplementary file 1 — Additional file 1. Sociodemographic and clinical data questionnaire. [file 12889_2020_9241_MOESM1_ESM.docx]

**Sociodemographic and clinical data questionnaire**

**Please check one box for each question where there are check boxes.**

**Age (years):**

- 31-40
- 41-50
- 51-60
- 61-70
- >70

**Sex:**

- Male
- Female

**Marital status:**

- Unmarried
- Married
- In a relationship
- Widow/widower

**Education:**

- Primary/junior high school
- Vocational
- Secondary
- Higher

**Employment status:**

- Student
- Unemployed
- Employed
- Retiree
- Pensioner

**Place of residence:**

- City with less than 50,000 population
- City with 50,000-200,000 population
- City with more than 200,000 population
- Village

**Disease duration (years):**

- ≤5 lat
- 6-10
- 11-15
- 16-20
- > 20

**Type of diabetes mellitus:**

- Type 1
- Type 2

**Treatment used:**

- Tablets only
- Insulin only
- Tablets + insulin

**Presence of diabetes complications** (select all that apply):

- Retinopathy
- Nephropathy
- Neuropathy
- Diabetic foot syndrome
